# High Resolution Micro-patterning of Stretchable Polymer Electrodes through Directed Wetting Localization

**DOI:** 10.1038/s41598-019-49322-7

**Published:** 2019-09-10

**Authors:** Sujie Kang, Bo-Yeon Lee, Sin-Hyung Lee, Sin-Doo Lee

**Affiliations:** Department of Electrical Engineering and Computer Science, Seoul National University, 1 Gwanak-ro, Gwanak-gu, Seoul, 08826 Republic of Korea

**Keywords:** Applied physics, Electronics, photonics and device physics

## Abstract

A microarray of conducting polymer electrodes with high resolution and high pattern-fidelity is developed on a stretchable substrate through the directed wetting localization (DWL) by the differential hydrophobicity. The large difference in the surface energy between the wetting and dewetting regions serves as the major determinant of the pattern resolution and the pattern-fidelity, yielding the full surface coverage in the stretchable electrode array (SEA) with 30 μm in width. The electrical characteristics of the SEA are well preserved under different types of elastic deformations. All-solution-processed polymer light-emitting diodes (except for the cathode) based on our patterned stretchable electrodes show no appreciable degradation of the performance under stretching. The DWL provides a simple and effective way of building up diverse stretchable electrical and optoelectronic devices in advanced wearable and bio-integrated electronics.

## Introduction

Stretchable electronics have drawn much attention for a wide range of applications from wearable devices to medical systems^1–9^. Unlike conventional electronics, stretchable electronics using a bendable, twistable and stretchable substrate can be easily implemented onto curved surfaces of a human body. For the realization of such devices, as a prerequisite, it is important to fabricate the high-resolution pattern of electrodes preserving the stretching capability. Until now, a variety of conducting materials such as graphene^6,10^, thermally evaporated metal^11^, carbon nanotubes^12–15^, metal nanowires^16^ and conducting polymers (CPs)^17–23^ have been widely used as stretchable electrodes. Among them, the CP is promising for a stretchable electrode owing to numerous advantages such as the mechanical flexibility, the cost-effectiveness, the biocompatibility, the solution processing capability and the robustness.

Considerable efforts have been made toward patterning the CPs on a stretchable substrate by photolithography^24,25^, screen printing^26,27^, inkjet printing^17,28^ and selective wetting-based patterning (SWP)^29^. Particularly, the SWP is a simple and cost-effective method for the large-area production of solution-processed patterns of the CPs^29–32^. Due to the intrinsically stronger hydrophobic nature of the stretchable substrate^33–36^ than typical rigid and flexible substrate^37,38^, however, the resolution of the CP patterns is quite limited to hundreds of micrometers in width^29^, not sufficient for stretchable electronic applications. Therefore, it is challenging to precisely localize the wetting regions for the construction of the CP patterns with the high accuracy.

**Figure 1:**
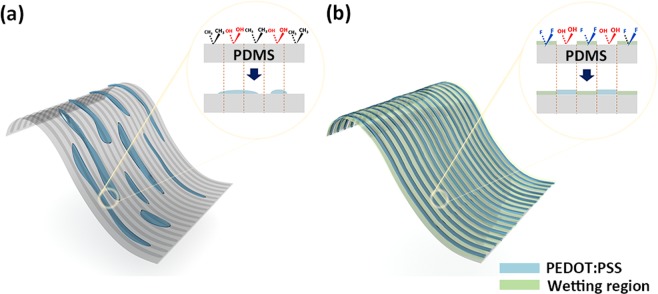
Schematic diagrams showing the concept of selective wetting (**a**) without and (**b**) with the DWL. The inset in each case shows the illustration of the chemical composition of the PDMS surface after the selective UVO treatment and resultant PEDOT:PSS pattern.

In this work, an array of stretchable polymer electrodes with high resolution and high pattern-fidelity was developed through the directed wetting localization (DWL). Here, a typical CP, poly(3,4-ethylenedioxythiophene):poly(styrenesulfonate) (PEDOT:PSS) was chosen for stretchable electrodes. Poly(dimethylsiloxane) (PDMS) was used as a stretchable substrate because of its considerably low value of the Young modulus, high transparency and biocompatibility. However, due to its hydrophobic nature, the PDMS shows a relatively small difference in the surface energy (SE) between the wetting and dewetting regions compared to the conventional substrates such as glass or plastic substrates. This intrinsic nature of the bare PDMS prevents from producing accurate electrode patterns on it through a conventional surface modification method as illustrated in Fig. 1(a). In contrast, the DWL utilizing the selective super-hydrophobic chemical modification through abundant fluorine on the dewetting region leads to the increase of the SE difference between the wetting and dewetting regions. As a result, the DWL enables to improve the pattern-fidelity of the PEDOT:PSS film as shown in Fig. 1(b). Through the DWL, being simple but accurate, the full coverage of the stretchable electrode microarray (SEA) with each pattern of 30 μm wide was achieved in the desired area. The SEA showed stable electrical characteristics under external deformations such as the cyclic tensile strain, twisting and stretching regardless of the pattern width. Using the stretchable polymer electrode as an anode, an all-solution-processed polymer light-emitting diode (S-PLED) was fabricated on the PDMS. The light emission from the S-PLED showed no appreciable degradation under the uniaxial strain of 20%.

## Results

### Dependence of PEDOT:PSS pattern-fidelity on selective surface modification

**Figure 2:**
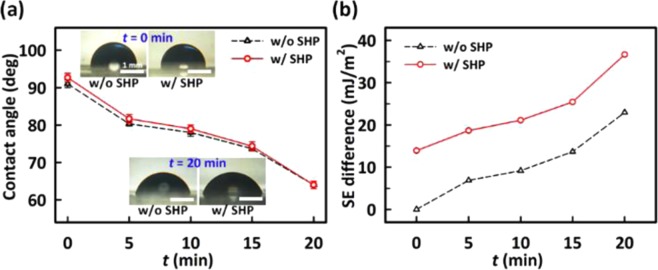
(**a**) The contact angles of water on the PDMS surfaces as a function of the UVO treatment time (*t*) and (**b**) the SE difference without (black triangles) and with (red circles) the SHP as a function of a function of *t*. The microscopic images in (**a**) show the corresponding water droplets on the surfaces at *t* = 0 and 20 min.

Let us first examine the differential hydrophobicity of the ultraviolet/ozone (UVO) treated regions in the super-hydrophobic polymer (SHP) and the PDMS. Two types of the substrates were prepared; one was the SHP-patterned PDMS and the other was the bare PDMS as a reference. Note that the selective UVO treatment through a photomask makes the treated region a rather hydrophilic nature of surface modification from the hydrophobic surface. In Fig. 2(a), as the duration time of the UVO treatment (*t*) increases from 0 to 20 min, the contact angle of water on the surfaces of the bare PDMS and the SHP-patterned PDMS decreases from 91° to 63° and 92° to 64°, respectively, in a similar fashion. The inset in Fig. 2(a) shows the corresponding microscopic images of water droplets on only the wetting region of the bare PDMS and that of the SHP-patterned PDMS at *t* = 0, 20 min for comparison. This indicates clearly that the strength of the hydrophobicity decreases with increasing *t*. The longer the duration time is, the less hydrophobicity is expected. However, above *t* = 20 min, the photomask in conformal contact with the surface of the wetting regions could not be detached from the substrate due to the strong adhesion between them. Note that the hydroxyl group density on the PDMS surface increases with the duration of the UVO treatment^39^. Such surface hydroxyl groups promoted on the wetting regions by the UVO yield the strong bonding of the PDMS with the photomask. Figure 2(b) shows the values of the difference in the SE between the wetting and dewetting regions in the two substrates. They were calculated from the measured contact angles using the Owens-Wendt method^40^ (see the Supplementary Information for details). It should be noted that at *t* = 20 min, the SE difference (36.64 mJ/m^2^) in the SHP-patterned PDMS is much larger than that of the reference substrate (22.96 mJ/m^2^). This is because the SE in the dewetting region of the SHP-patterned PDMS is much lower than that of the reference (see Fig. S1), although the values of the SE in the wetting region in both cases are similar to each other. It means that the DWL is indeed capable through the SHP patterns on the PDMS.

**Figure 3:**
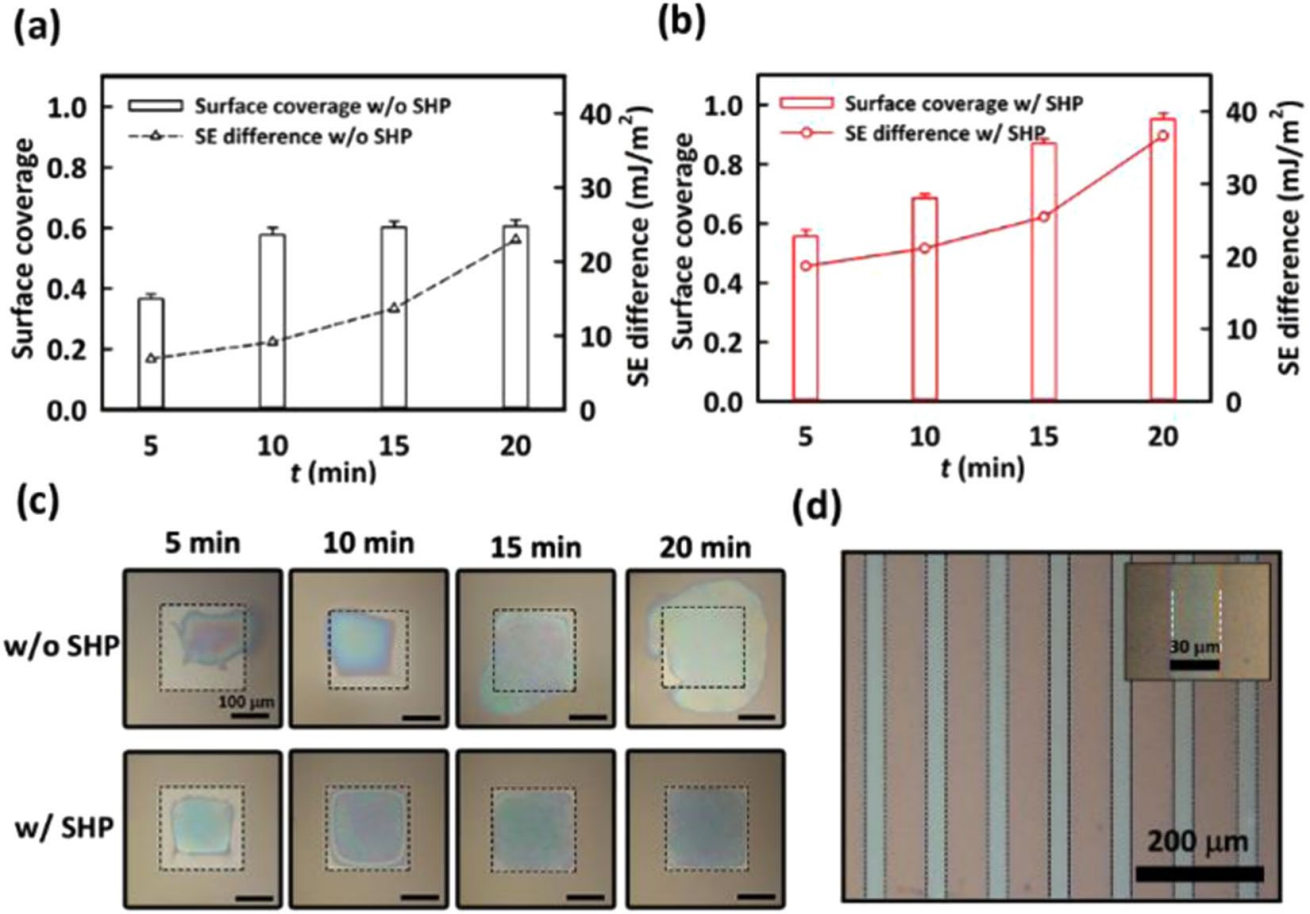
The surface coverage of the PEDOT:PSS patterns and the SE difference (**a**) without and **(b)** with the SHP patterns as a function of *t*. (**c**) The corresponding microscopic images of the PEDOT:PSS patterns (top) without and (bottom) with the SHP patterns. (**d**) The microscopic images of the PEDOT:PSS film with each pattern of 30 μm wide.

In evaluating the pattern-fidelity of the PEDOT:PSS through the DWL, we measured the surface coverage, defined as the ratio of the effective area of the PEDOT:PSS to the whole area of the wetting region of the square pattern with each side of 200 μm long. The overflow of the PEDOT:PSS into the dewetting region was considered to be an error region, being excluded from the effective area of the PEDOT:PSS in calculation of the surface coverage. Compared to the limited surface coverage (up to about 0.6) of the PEDOT:PSS on the reference substrate as shown in Fig. 3(a), the surface coverage of the PDMS with the SHP patterns reached at about 0.96 from 0.58 with increasing the SE difference as shown in Fig. 3(b). Note that the SE difference is critical for the surface coverage but not the magnitude of the SE itself. Figure 3(c) shows the corresponding microscopic images of the PEDOT:PSS patterns on the two surfaces as *t* increased from 0 to 20 min, one without the SHP (top) and the other with the SHP (bottom). Clearly, the PEDOT:PSS pattern was well defined on the surface with the SHP at *t* = 20 min. Figure 3(d) shows the full coverage of the PEDOT:PSS in each line pattern of 30 μm wide. The expanded image as shown in the inset of Fig. 3(d) clearly shows the high pattern-fidelity of the PEDOT:PSS along the edges of the groove. It can be then concluded that in addition to the increase of the SE in the wetting region, the SE difference between the wetting region and the dewetting region plays an important role in achieving high resolution and high pattern-fidelity of the PEDOT:PSS on the stretchable PDMS substrate^41,42^.

### Electrical and mechanical characteristics of the SEA

**Figure 4:**
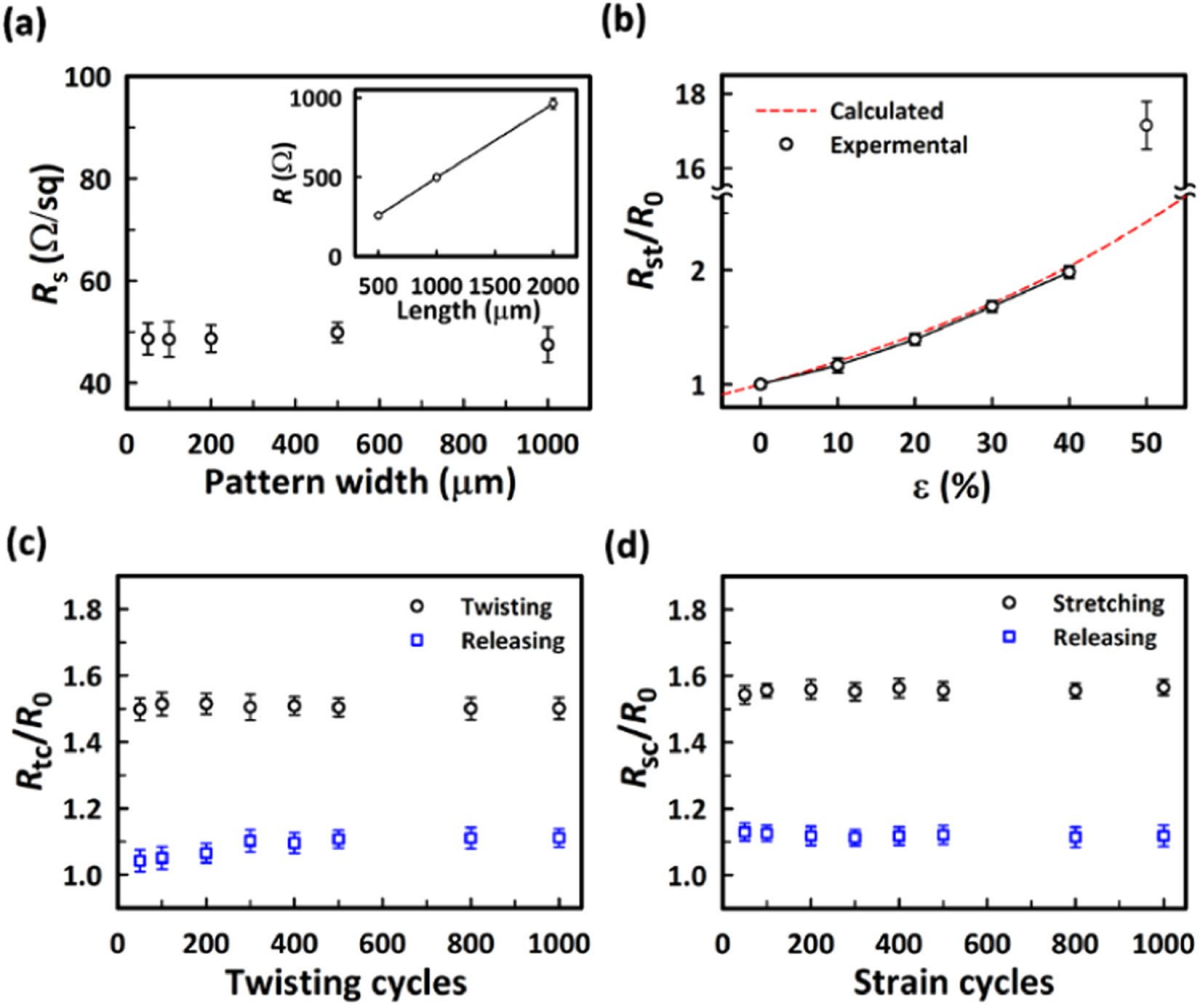
(**a**) The sheet resistance (*R*_s_) of the PEDOT:PSS patterns with different widths. The inset of (a) shows the resistance of the PEDOT:PSS patterns with different lengths. (**b**) *R*_st_/*R*_o_ of the PEDOT:PSS patterns as a function of the strain (ε) up to 50%. (**c**) *R*_tc_/*R*_o_ and (**d)** *R*_sc_/*R*_o_ of the PEDOT:PSS patterns under cyclic twisting of the angle of 180° and stretching of 25% for 1000 cycles, respectively.

In Fig. 4(a), the sheet resistance (*R*_s_) for a single line pattern with the length of 2000 μm and the width varying from 50 μm to 1000 μm in our SEA was measured as a function of the pattern width. In general, *R*_s_ decreases with increasing the thickness of the PEDOT:PSS pattern^43^. In our case, the PEDOT:PSS thickness was about 180 nm which was obtained through spin-coting by three times. The average value of *R*_s_ of the pattern remained fairly constant to be 48.87 ± 2.77 Ω/sq irrespective the pattern width from 50 μm to 1000 μm. This is indeed comparable to the range of *R*_s_ (below 50 Ω/sq) applicable for a variety of wearable electronic and optoelectronic devices^44^. As expected, for given pattern width (100 μm), the resistance of a line pattern increased linearly with the pattern length as shown in the inset of Fig. 4(a); again, the average value of *R*_s_ was nearly independent of the pattern length, 48.91 ± 1.72 Ω/sq from 500 μm to 2000 μm in length.

We evaluated the mechanical stability of the SEA composed of three patterns in stripe (each of 2000 μm long and 100 μm wide) on the PDMS substrate. The change of the total resistance of the three stripes was measured under different types of elastic deformations such as the tensile strain, the cyclic twisting, and the cyclic stretching. The initial resistance (*R*_0_) under no deformation was 982.7 Ω. Figure 4(b) shows the change of the resistance (*R*_st_) under the tensile strain (ε) in relative to *R*_0_ as a function of ε. The theoretical values of *R*_st_, calculated from the equation of resistance using the Poisson’s ratio^45^, were also presented (a dashed line) in Fig. 4(b) (see the Supplementary Information for the detailed description). The experimental results were found to be in good agreement with the calculated values in the range of ε up to 40%. Above ε = 50%, defects such as cracks were developed in the PEDOT:PSS electrodes so that the loss of the electrical contact occurred^45,46^. In fact, ε ~ 40% is a typical range of stretching corresponding to the range of motion in human joints^47^.

We also estimated the durability of the SEA under the cyclic elastic deformations of stretching and twisting. In the twisting test at the twist angle of 180°, the resistance was increased by about 50% as shown in Fig. 4(c). Such increase of the resistance of the SEA is mainly attributed to the dimensional changes of the PEDOT:PSS under the strain of twisting. After releasing from the first twisting cycle, the resistance was slightly increased by about 9% but remained fairly constant irrespective of further twisting cycles. In Fig. 4(d), the change of the resistance (*R*_sc_) under the cyclic stretching relative to *R*_0_ between ε = 0 and ε = 25% was shown as a function of the number of the cycles up to 1000. Under the stretching of ε = 25%, the resistance was increased by about 55%. After restoring from the first stretching cycle, the resistance was increased by about 13%. However, essentially no further increase of the resistance was observed during repeated stretching up to 1000 cycles.

As an applicable example, our SEA was applied to the stretchable interconnects (as shown in Supplementary Fig. S2) where it was composed of three PEDOT:PSS patterns in stripe (each of 200 μm wide and 2000 μm long) to interconnect 10 commercial red LEDs in series. In the twisting test (see Fig. S2(a)), the electrical characteristics of the stretchable interconnects were unchanged at the twist angle of 120°. For the case of stretching (see Fig. S2(b)), during stretching the SEA wrapped around a finger, the electrical characteristics of the stretchable interconnects were well preserved at ε = 20%. No appreciable degradation of the luminance was observed, consistent with the results for the resistance under stretching in Fig. 4(d). Note that an elastomeric PDMS film (about 100 μm thick) with the PEDOT:PSS electrode was maintained in conformal contact with the finger under bending. This indicates that our SEA shows the good stability of the electrical performance against repeated elastic deformations.

### Light-emitting properties of the elastomeric S-PLEDs

**Figure 5:**
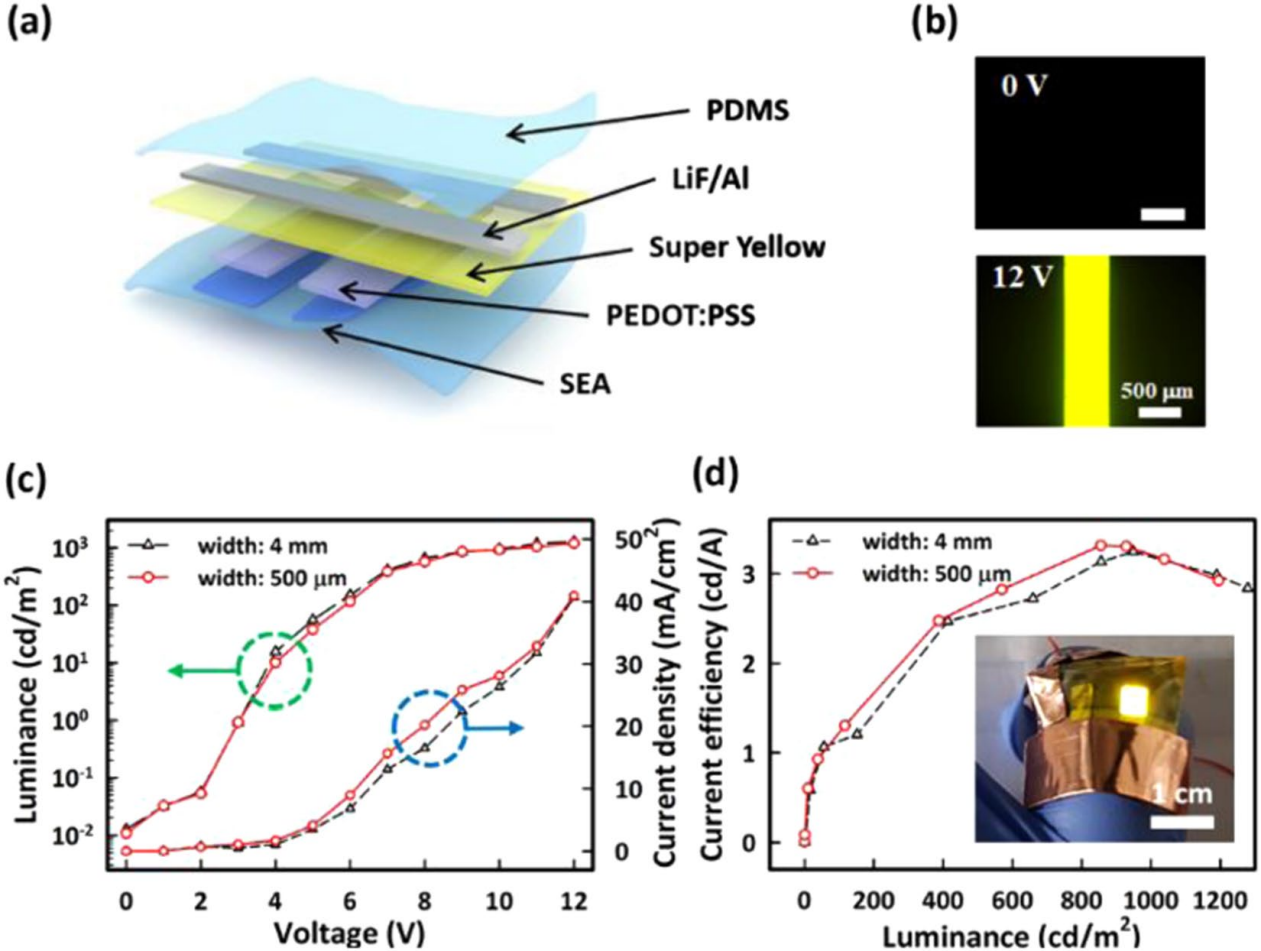
Schematic illustration and the emission characteristics of the S-PLED with the stretchable anode. (**a**) The structure of the S-PLED. (**b**) The microscopic images showing the light emission of the S-PLED with the SEA of 500 μm wide at *V*_d_ = 0 and 12 V. (**c**) Current density and luminance of the S-PLED with 4 mm wide (black triangles) and a stretchable anode of 500 μm wide (red circles) after stretching of ε = 20% as a function of *V*_d_. (**d**) Current efficiency of the S-PLED as a function of the luminance. The inset in (**d**) shows the photograph of the S-PLED (the anode of 4 mm wide), uniformly emitting light under stretching of ε = 20%.

We also fabricated the S-PLED with the stretchable anode on an elastomeric substrate. The SEA consisting of three PEDOT:PSS patterns in stripe (each of 500 μm wide and 2000 μm long) was used as the stretchable anode. As shown in Fig. 5(a), the S-PLED was made of five layers of PEDOT:PSS anode/PEDOT:PSS/PDY-132/LiF/Al, produced through three solution-processes for organic layers (the anode, the hole transport layer, and the emission layer) in sequence and vacuum deposition for the cathode at the pressure of 2.0 × 10^−6^ torr. Figure 5(b) shows the light emission of the S-PLED at two driving voltages (*V*_d_) of 0 and 12 V. Before and after stretching at ε  = 20%, the current density and the luminance of the S-PLED with the stretchable anode of 500 μm wide were shown as a function of *V*_d_ (as shown in Fig S3). Before stretching, the turn-on voltage of the S-PLED was 5 V. The maximum luminance was 1195.1 cd/m^2^ at 12 V where the current density was 40.7 mA/cm^2^. After stretching, the luminance and the current density of the S-PLED at 12 V were 1055.4 cd/m^2^ and 40.3 mA/cm^2^, respectively. Those values were slightly decreased due to the slight increase of *R*_s_ of the PEDOT:PSS anode under stretching at ε = 20%. The corresponding current efficiency was also shown as a function of the luminance (see Fig. S3). As clearly seen from Fig. 5(c,d), for the case of the SEA consisting of two PEDOT:PSS patterns in stripe (each of 4 mm wide and 20 mm long), no appreciable degradation of the emission characteristics of the S-OLED were observed irrespective of the pattern width of the stretchable anode.

## Discussion

We presented the concept of the DWL based on the differential hydrophobicity to construct the SEA with high resolution and high pattern-fidelity. The DWL was indeed capable of fabricating the SHP patterns by means of the SE difference between the wetting and dewetting regions on the elastomeric PDMS, allowing for the SEA with the full coverage of the PEDOT:PSS in the wetting region of 30 μm wide. We demonstrated that the electrical characteristics of the SEA for stretchable interconnects among a number of the LEDs and the stretchable anode of the S-PLED were well preserved under different types of the elastic deformations such as the tensile strain, the cyclic twisting, and the cyclic stretching. Our DWL approach to the production of the high-resolution SEA is widely applicable for next-generation wearable and bio-implemented electronics.

## Methods

### Fabrication of PEDOT:PSS patterns on PDMS

For the detachment of the SEA from a solid substrate (glass), a hydrophobic polymer (Novec^TM^ EGC-1700, 3 M) dissolved in a fluorinated solvent (Novec^TM^ HFE-7100, 3 M) at the concentration of 2%, was first spin-coated at 2000 rpm for 30 s on a cleaned glass substrate. An elastomer mixture of the PDMS (Sylgard 184, Dow Corning), the PDMS base and a curing agent (Sylgard 184) at 10:1 ratio by mass, was then poured onto the fluorinated polymer layer to prepare a stretchable PDMS film. The substrate was subsequently placed in a vacuum oven for 30 min to eliminate air bubbles in the mixture and annealed at 70 °C for 2 h. The thickness of the PDMS film was about 100 μm. A super-hydrophobic polymer (SHP) (DS-1120, Harves Co., Ltd), having lower SE than the PDMS, was finally spin-coated on the top of the PDMS film at 4000 rpm for 30 s and annealed at 80 °C for 30 min. Before the selective surface modification on two different types of the PDMS substrates (one without and the other with the DWL), the ultraviolet/ozone (UVO) treatment was carried out on the surface of the SHP film coated on the PDMS through a photomask for the complete etching of the treated regions of the SHP film; the SHP has the hydrophobicity higher than the PDMS and easily etched by the UVO treatment (see Fig. S4). In order to etch out the SHP and to produce the surface modification, the UVO treatment was performed at the intensity of 28 mW/cm^2^ (AH-1700, Ahtech LTS Co., Ltd.). The SHP patterns in the PDMS surface, characterized using the field emission-scanning electron microscope (FE‐SEM, S‐4800; Hitachi), were well-defined (see Fig. S5). The duration time of the UVO treatment was varied from 0 to 20 min to vary the strength of the hydrophobicity. During the entire UVO treatment, a conformal contact of the photomask with the substrate was maintained. An aqueous solution of PEDOT:PSS (Celvios™ PH1000, Heraeus) was spin-coated onto the substrate at 2000 rpm for 30 s. The substrate with the PEDOT:PSS layer was annealed at 120 °C for 15 min to evaporate any remaining solvent. Note that the PEDOT:PSS solution contained 5 wt. % of ethylene glycol and 0.5 wt. % of fluorosurfactant Zonyl (Capstone^®^ FS-30, Dupont^TM^) to improve its conductivity and wetting on the hydrophobic PDMS surface, respectively^48,49^.

### Optical, electrical, and mechanical characterization of the SEA

The surface pattern of the PEDOT:PSS layer on the wetting region was characterized using an optical polarizing microscope (Optiphot-Pol, Nikon). An image processing software (Image J) was used for measuring the area fraction of the PEDOT:PSS film in the wetted region from the microscopic image. The value of *R*_s_ of the PEDOT:PSS film was measured using the four-point probe method and calculated from the equation of resistance. The magnitude of the mechanical stretching of the SEA was determined using a home-made stretching stage. The repetitive stretching test was performed at a frequency of 0.5 Hz for 1000 cycles. The dynamic cyclic test was performed at a frequency of 0.25 Hz for 10 min. The electrical interconnects among red light-emitting diodes (LEDs) (BL-B5134(333GD), BRIGHT LED) by the SEA was directly monitored by the application of the voltage from a DC Regulated power supply (QJE).

### Fabrication of S-PLED with stretchable anode

The PEDOT:PSS patterns in stripe (each of 180 nm wide) for use as an anode were prepared on the PDMS film through the DWL. A hole transport layer (HTL) of the PEDOT:PSS (Celvios™ Al 4083, Heraeus) was spin-coated on the PEDOT:PSS anode at 2000 rpm for 30 s and annealed at 120 °C for 15 min. The thickness of the HTL was about 50 nm. For an emission layer (EML), a solution of the Super Yellow polymer (PDY-132, Sigma-Aldrich) (80 nm in thick), dissolved in anhydrous toluene at 0.6 wt. %, was spin-coated at 2000 rpm for 60 s on the HTL and annealed at 90 °C for 60 min in a glove box system with low moisture and oxygen content. A 0.5 nm-thick layer of lithium fluoride (LiF) and a 100 nm-thick layer of aluminum (Al) were thermally deposited through a shadow mask at the rate of 0.1 Å/s and 5.0 Å/s in a vacuum chamber under 2.0 × 10^−6^ torr, respectively. The PDMS film of 100 μm thick was prepared for use as the passivation layer on the S-PLED. The current density-voltage-luminance (J-V-K) characteristics of the S-PLED were measured using a spectrometer (CS-2000, Konica Minolta) in conjunction with a source meter (2636A, Keithley instruments, Inc.).

## Supplementary information

Supplementary Information
