# Supplementary material for: High Resolution Micro-patterning of Stretchable Polymer Electrodes through Directed Wetting Localization
Source: Sci Rep. 2019 Sep 10;9:13066. doi: 10.1038/s41598-019-49322-7 (PMC6737050; doi:10.1038/s41598-019-49322-7)
Supplement: Supplementary file 1 — Supplementary Information [file 41598_2019_49322_MOESM1_ESM.pdf]

Supplementary Information

High Resolution Micro-patterning of Stretchable Polymer Electrodes through Directed Wetting Localization

*Sujie Kang, Bo-Yeon Lee, Sin-Hyung Lee, Sin-Doo Lee**

1. Calculation of the resistance under strain relative to the intrinsic resistance

For a conducting film, its intrinsic resistance (R_0) can be expressed as

$$R_0 = \frac{1}{\sigma}\frac{l}{wt} \quad , \tag{1}$$

where the conductivity, the length, the width, and the thickness are denoted by σ, *l*, *w*, and *t*, respectively, under no strain. If the conducting film experiences certain dimensional changes, the resultant resistance under the strain (R_{st}) is given by

$$R_{st} = \frac{1}{\sigma}\frac{l(1+\varepsilon')}{w(1-\nu_f\varepsilon')t(1-\nu_s\varepsilon')} \quad , \tag{2}$$

where ν_f and ν_s are the Poisson's ratio of the film and that of the substrate, respectively. The fractional strain is assumed to be ε' = ε / 100. Under stretching, the increase in length is described in terms of (1 + ε'). Accordingly, the decrease in the width and that in the thickness are (1 - ν_sε') and (1 - ν_fε'), respectively. As shown in Fig. 4(b), based on these changes, a theoretical plot of R_{st}/R_0 as a function of ε can be expressed as

$$\frac{R_{st}}{R_0} = \frac{1+\varepsilon'}{(1-\nu_f\varepsilon')(1-\nu_s\varepsilon')} \quad . \tag{3}$$

Here, the reported value of ν_f of the PEDOT:PSS is 0.35, and the value of ν_s of the PDMS is 0.5.

2. Calculation of the surface energy using the Owens-Wendt method

Thermodynamic wetting can be described in terms of the well-known Young equation[S1],

$$\cos\theta\gamma_{lv} = \gamma_{sv} - \gamma_{sl} - \pi_e, \quad (1)$$

where γ_{lv} and γ_{sv} are the free energy of the liquid and that of the solid against the vapor, respectively. Here, γ_{sl} represents the interfacial energy between the liquid and the solid. The contact angle between the liquid and the solid and the equilibrium pressure of the absorbed vapor of the liquid on the solid are denoted by θ and π_e, respectively. Under the condition that γ_{lv} corresponds to the surface tension of complete wetting (γ_c), namely, $\pi_e = 0$ when $\theta = 0$, Eq. (1) can be written as

$$\cos\theta\gamma_{lv} = \gamma_c = \gamma_{sv} - \gamma_{sl}. \quad (2)$$

Moreover, following the Fowkes equation in the theoretical consideration of the total free energy at a surface[S2],

$$\gamma_{sl} = \gamma_{sv} + \gamma_{lv} - 2\sqrt{\gamma_s^d\gamma_l^d} - 2\sqrt{\gamma_s^h\gamma_l^h}\ , \quad (3)$$

where the surface energy of water $\gamma_{lv} = \gamma_l^d + \gamma_l^h$ (the superscripts *d* and *h* refer to the dispersion force and the hydrogen bonding, respectively). This can be expressed alternatively as

$$\gamma_{sl} = \left(\sqrt{\gamma_s^d} - \sqrt{\gamma_l^d}\right)^2 + \left(\sqrt{\gamma_s^h} - \sqrt{\gamma_l^h}\right)^2. \quad (4)$$

Combining Eqs. (2) and (4), the relationship between the contact angle and all the relevant surface tensions is given by

$$1 + \cos\theta = 2\sqrt{\gamma_s^d}\left(\frac{\sqrt{\gamma_l^d}}{\gamma_{lv}}\right) + 2\sqrt{\gamma_s^h}\left(\frac{\sqrt{\gamma_l^h}}{\gamma_{lv}}\right), \quad (5)$$

where γ_s^d and γ_s^h represents the solid case. Using the above Eq. (5), we analyzed our experimental data. The results were shown in Figs. 2 and 3.

References

[S1] Owens, D. K. & Wendt, R. C. Estimation of the surface free energy of polymers. *J. Appl. Polym. Sci.* **13**, 1741–1747 (1969).

[S2] Fowkes, F. M. Attractive forces at interfaces. *Ind. Eng. Chem.* **56**, 40-52 (1964).

3. Supplementary Figure S1

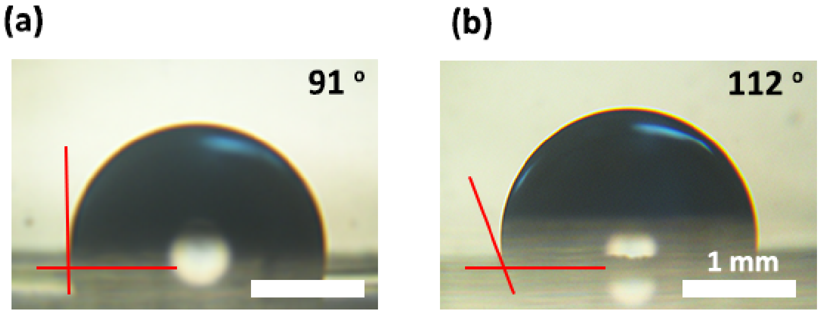

Figure S1. The microscopic images of water droplets on the surfaces of the dewetting region on (a) the bare PDMS and (b) the SHP-patterned PDMS.

4. Supplementary Figure S2

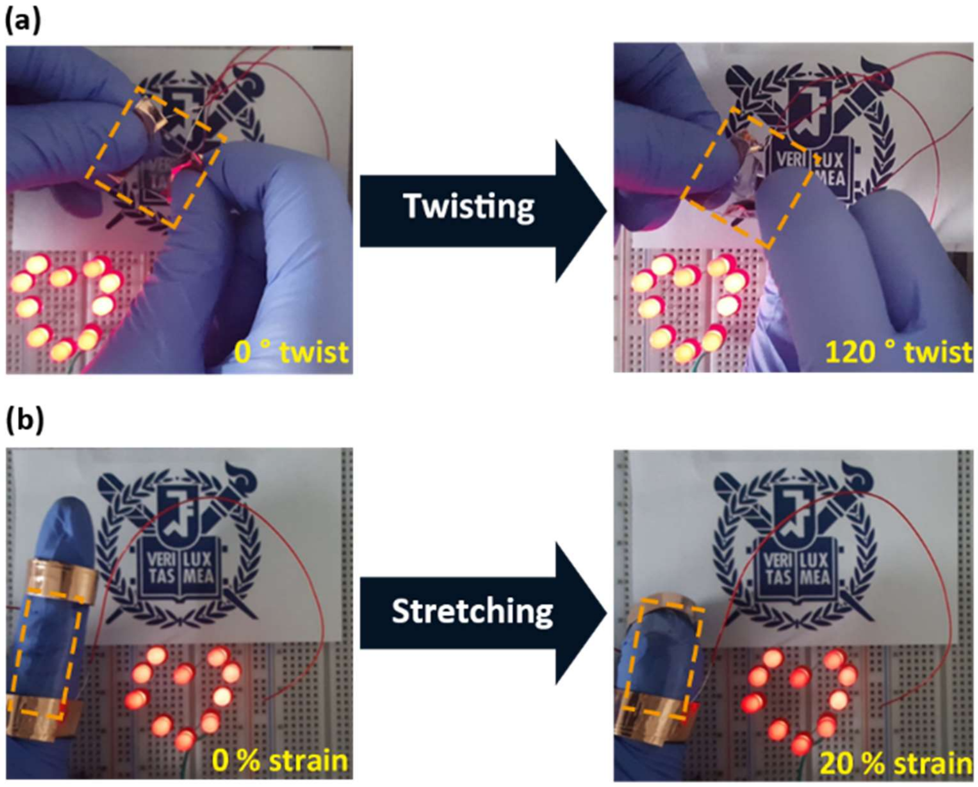

Figure S2. Applications of the SEA for the stretchable interconnects.

The photographs of 10 LEDs, interconnected using the SEA, showing no appreciable degradation of the luminance under (a) twisting (the angle up to 120°) and (b) stretching (the strain up to 20 %). The rectangular regions enclosed by orange dashed lines represent the stretchable interconnects.

5. Supplementary Figure S3

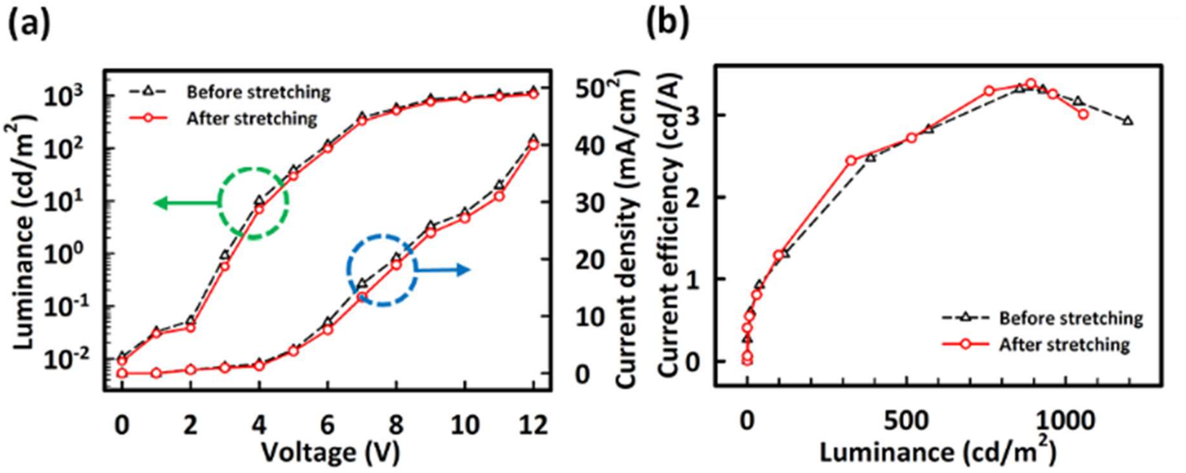

Figure S3. The light emission characteristics of the S-PLED with the stretchable anode of 500 μm wide depending on the stretching. (a) Current density and luminance of the S-PLED before (black triangles) and after (red circles) stretching of ε = 20 % as a function of V_d. (b) Current efficiency of the S-PLED as a function of the luminance.

6. Supplementary Figure S4

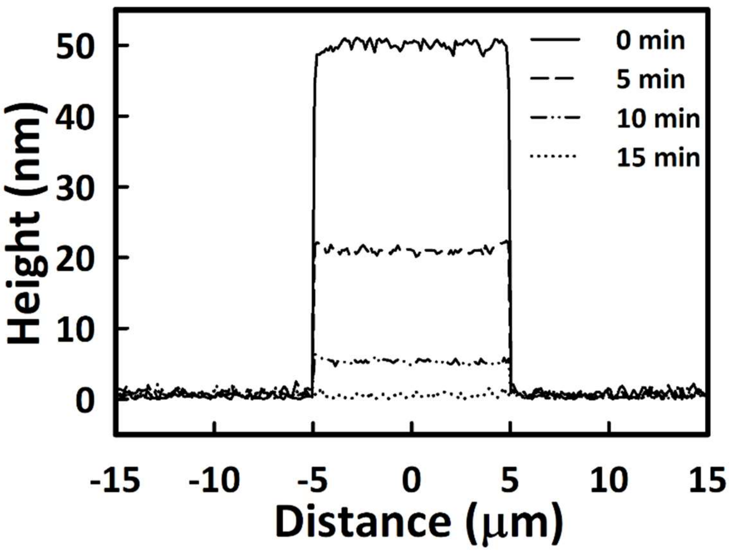

Figure S4. The geometrical profiles across the pattern of the SHP as a function of the UVO treatment time after etching.

7. Supplementary Figure S5

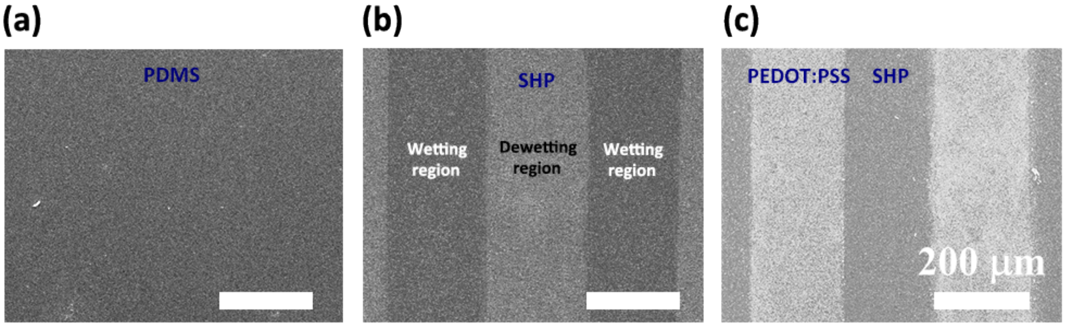

Figure S5. The FE-SEM images of the surfaces of (a) the bare PDMS and (b) the SHP-patterned PDMS at t = 20 min. (c) The FE-SEM image of the PEDOT:PSS patterns formed on the SHP-patterned PDMS. The width of each pattern is 200 μm.
